# Histopathological changes of organ dysfunction in sepsis

**DOI:** 10.1186/s40635-019-0236-3

**Published:** 2019-07-25

**Authors:** Antonio M. Garofalo, Marta Lorente-Ros, Gesly Goncalvez, Demetrio Carriedo, Aída Ballén-Barragán, Ana Villar-Fernández, Óscar Peñuelas, Raquel Herrero, Rosario Granados-Carreño, José A. Lorente

**Affiliations:** 1 Hospital Universitario de Getafe, Madrid, Spain; 2 Universidad Europea de Madrid, Madrid, Spain; 3 Hospital Universitario La Paz, Madrid, Spain; 4 CIBER de Enfermedades Respiratorias, Madrid, Spain

**Keywords:** Sepsis, Septic shock, Organ dysfunction, Kidney, Liver, Brain, Histopathology, Autopsy, Light microscopy

## Abstract

**Background:**

Sepsis is a highly lethal disorder. Organ dysfunction in sepsis is not defined as a clinicopathological entity but rather by changes in clinical, physiological, or biochemical parameters. Pathogenesis and specific treatment of organ dysfunction in sepsis are unknown. The study of the histopathological correlate of organ dysfunction in sepsis will help understand its pathogenesis.

**Methods:**

We searched in PubMed, EMBASE, and Scielo for original articles on kidney, brain, and liver dysfunction in human sepsis. A defined search strategy was designed, and pertinent articles that addressed the histopathological changes in sepsis were retrieved for review. Only studies considered relevant in the field were discussed.

**Results:**

Studies on acute kidney injury (AKI) in sepsis reveal that acute tubular necrosis is less prevalent than other changes, indicating that kidney hypoperfusion is not the predominant pathogenetic mechanism of sepsis-induced AKI. Other more predominant histopathological changes are apoptosis, interstitial inflammation, and, to a lesser extent, thrombosis. Brain pathological findings include white matter hemorrhage and hypercoagulability, microabscess formation, central pontine myelinolysis, multifocal necrotizing leukoencephalopathy, metabolic changes, ischemic changes, and apoptosis. Liver pathology in sepsis includes steatosis, cholangiolitis and intrahepatic cholestasis, periportal inflammation, and apoptosis. There is no information on physiological or biochemical biomarkers of the histopathological findings.

**Conclusions:**

Histopathological studies may provide important information for a better understanding of the pathogenesis of organ dysfunction in sepsis and for the design of potentially effective therapies. There is a lack of clinically available biomarkers for the identification of organ dysfunction as defined by the histological analysis.

## Background

### Why is it interesting to study the histopathology in sepsis-induced organ dysfunction?

Sepsis is a highly lethal disorder that is responsible for the death of over 200,000 people annually in the USA alone [1]. More deaths are due to sepsis and septic shock than myocardial infarction, even in the Western world [2].

Unlike classical disease entities, such as cancer and infectious or immunological conditions, organ dysfunction in the context of sepsis and acute respiratory failure is not defined as a clinicopathological entity but rather described by nonspecific clinical changes (i.e., level of consciousness), physiological alterations (hypotension, decreased urine output) or serum biomarkers of organ function (i.e., serum concentration of bilirubin or creatinine, blood platelet count). However, the pathological correlate of those clinical, physiological, or biochemical changes characteristic of organ dysfunction or, even more importantly, the clinical, physiological, or biochemical changes corresponding to pathological abnormalities in patients with sepsis, are to a great extent not well defined.

The aim of this study is to summarize the histopathological changes in the kidney, brain, and liver associated with sepsis-induced organ dysfunction in humans. The description of these pathological changes is of particular relevance in the face of conditions whose pathogenesis is not well defined and for which specific treatments are not available. Knowledge of pathological findings associated with sepsis could help with a better understanding of pathogenic mechanisms, a more precise knowledge on the reversibility of organ dysfunction, and a more effective design of potentially effective treatments.

In this review we examine the most important studies on the pathological changes associated with organ dysfunction induced by human sepsis, focusing on the kidney, brain, and liver. Lung pathology in patients with ARDS has been recently reviewed by us and others [3–13]. Results from experimental studies in animal models are used to enlighten the interpretation of findings from human studies. Based on the description of those findings, we speculate on pathogenic mechanisms involved in sepsis-induced organ dysfunction and on potentially effective therapies.

## Methods

A retrospective review of articles was done, using the databases PubMed, EMBASE, and Scielo. For the search, we used the keywords “sepsis” AND “pathology” OR “histology” AND “kidney” OR “brain” OR “liver”. We only included articles that studied human subjects with the diagnosis of sepsis or septic shock. Non-original studies were excluded. Articles in a language different from English were excluded. Articles whose title, keywords, or aim did not address the histopathological changes in sepsis of the kidney, brain, or liver were excluded. Articles that did not include a thorough clinical description of patients or a detailed description of the pathologic examination of biopsies were considered non-relevant. We included 78 articles published from 1962 to 2017.

Given the extended period of time that the articles cover, the interpretation of the herein reported histopathological findings should consider that the changing definition of sepsis in time could have an impact on the characteristics of the patients included in the different studies.

### Acute kidney injury in sepsis

Acute kidney injury (AKI) is common in critically ill patients and is independently associated with an increased mortality rate, and sepsis is its most common risk factor [14, 15]. The knowledge of the histopathology of sepsis-induced organ dysfunction is particularly important in the case of the kidney, as the presence of certain changes may support the implementation of specific therapies. For instance, changes indicative of acute tubular necrosis (ATN) may indicate that the role of hypoperfusion during the early phase of sepsis is predominant and thus more aggressive fluid resuscitation could be indicated to treat or prevent AKI. On the other hand, a predominance of inflammatory changes with a low incidence of ATN would point at a more important role of inflammation in AKI, and anti-inflammatory therapies could be more effective to treat or prevent sepsis-induced AKI.

ATN in the context of systemic hypotension and renal hypoperfusion has been generally accepted as the characteristic kidney pathological change in sepsis [16]. However, studies on kidney pathology in sepsis fail to reveal a high prevalence of ATN as a histological finding.

Mustonen and colleagues published the results of kidney tissue studies obtained by renal biopsy in 57 patients dying with sepsis, shock, or hypovolemia [17]. By light microscopy, nonspecific tubulointerstitial renal changes were the predominant histopathologic finding. In total, 82% of specimens showed acute tubulointerstitial nephropathy, whereas 7% showed acute glomerulonephritis, 3.5% showed acute pyelonephritis, and only four (7%) cases showed classic histopathologic findings consistent with ATN.

Hotchkiss and colleagues did a postmortem study of 12 patients with sepsis and AKI and found ATN in only 1 patient (5%) [18].

In the retrospective analysis by Diaz de Leon et al. [19] renal biopsies were performed in 40 septic patients with AKI. They found that 11 patients (27.5%) had nonspecific tubular or glomerular damage; 9 cases (22.5%) had evidence of vascular involvement; and 20 (50%) had ATN. Sato and colleagues studied postmortem 6 patients with sepsis and AKI [20] and found in 5 evidence of mild nonspecific general cell injury. Only 1 patient (17%) had evidence of ATN. In two single case reports in which a renal biopsy was done in a patient with clinical AKI, no ATN was found [21, 22].

In a systematic review of human studies published to that date, Langerberg et al. [23] found that of the 417 septic patients included in these six reviewed studies [17–22], 184 (44%) presented evidence of AKI, and 117 had histopathologic specimens available for evaluation, 26 of whom (22%) presented ATN in histological examination.

Lerolle et al. [24] studied renal biopsy specimens obtained from 19 consecutive patients who died of septic shock. In comparison with the control groups (8 patients who died of trauma at the scene and 9 ICU patients dying without sepsis) all patients presented acute tubular lesions; intense infiltration by leukocytes, mainly monocytic, in glomeruli and interstitial capillaries; and tubular cell apoptosis (in 2.9% of tubular cells, by the identification of apoptotic bodies, TUNEL, and activated caspase-3 staining. Arteriolar/arterial thromboses were observed in only 4 of 19 patients, without relationship with presence of disseminated intravascular coagulation. The intensity of acute tubular lesions correlated with blood lactate concentration. It has to be noted that these patients presented AKI of special severity as they were all anuric.

Thus, the findings in this study of different degrees of acute tubular lesions, intense infiltration of glomeruli, interstitial capillaries and occasionally tubular lumens by leucocytes and apoptosis of tubular cells, and thrombotic lesions [24] suggest important roles for inflammation and apoptosis, as well as, although to a lesser degree, for hypoperfusion and coagulation, in the pathogenesis of sepsis-induced AKI.

A limitation of this [24] and other studies is the control group used for comparison. Indeed, that the reported findings in patients with septic shock are attributable to sepsis, that is, they are not present in patients with shock of non-septic origin, remains to be proven.

In a postmortem study of renal changes in patients with sepsis, using kidneys from 20 trauma patients and eight patients with cancer as controls, Takasu et al. [25] found that 3 of 20 specimens showed focal injury in approximately 1% of renal cortical tubules [24]. Focal acute tubular injury was present in 78% of septic kidneys, occurring in 10.3 ± 9.5% and 32.3 ± 17.8% of corticomedullary-junction tubules by conventional light microscopy and Kim-1 immunostains, respectively. Electron microscopy revealed increased tubular injury in sepsis, including hydropic mitochondria and increased autophagosomes. The authors concluded that renal tubular injury is common but presents focally, that most renal tubular cells appear normal, and that the degree of cell injury identified does not account for severity of sepsis-induced organ dysfunction. This study [25] is in contrast with the study by Lerolle et al. [24], which found that tubular cell apoptosis and infiltration of mononuclear leukocytes were common, whereas Takasu et al. [25] did not find a predominant role of apoptosis in sepsis-induced AKI. Different detection methods could explain this discrepancy.

Renal histopathological changes in humans can be better interpreted in light of studies in experimental models. In a recent systematic review of histopathological changes in animal models of sepsis [26], in 1059 animals studied in 102 studies, only 53 (5.0%) did not have any renal histopathologic changes. ATN was reported in 184 (17.4%), only in animals with low cardiac output and diminished renal blood flow. Nonspecific changes were also observed: vacuolization of tubular cells in 423 (39.9%), loss of brush border in 250 (23.6%), and tubular cell swelling in 243 (22.9%). Of note, in the 21 studies (170 animals) that analyzed the presence of apoptosis, tubular cell apoptosis was found in 158 animals (92.9%).

Therefore, in general, pathological evaluation of the kidney in sepsis most frequently revealed non-specific changes, and in line with previous studies ATN was found in only one fifth of cases, and mostly in models with low cardiac output. This finding suggests that hypoperfusion, although it may have a role in the pathogenesis of sepsis-induced AKI in a certain proportion of cases, is not always the predominant etiologic factor. This concept is supported by the finding that septic AKI can develop with an increased or maintained renal blood flow [27, 28]. Importantly, tubular cell apoptosis was found in more than 90% of animals in which this abnormality was searched. Tubular cell apoptosis has been recognized, as in the animal models, a prominent feature of human septic AKI [29, 30]. Apoptosis may result from inflammation, oxidative stress, or ischemia. However, apoptosis may occur in the presence of normal blood flow [31].

**Fig. 1:**
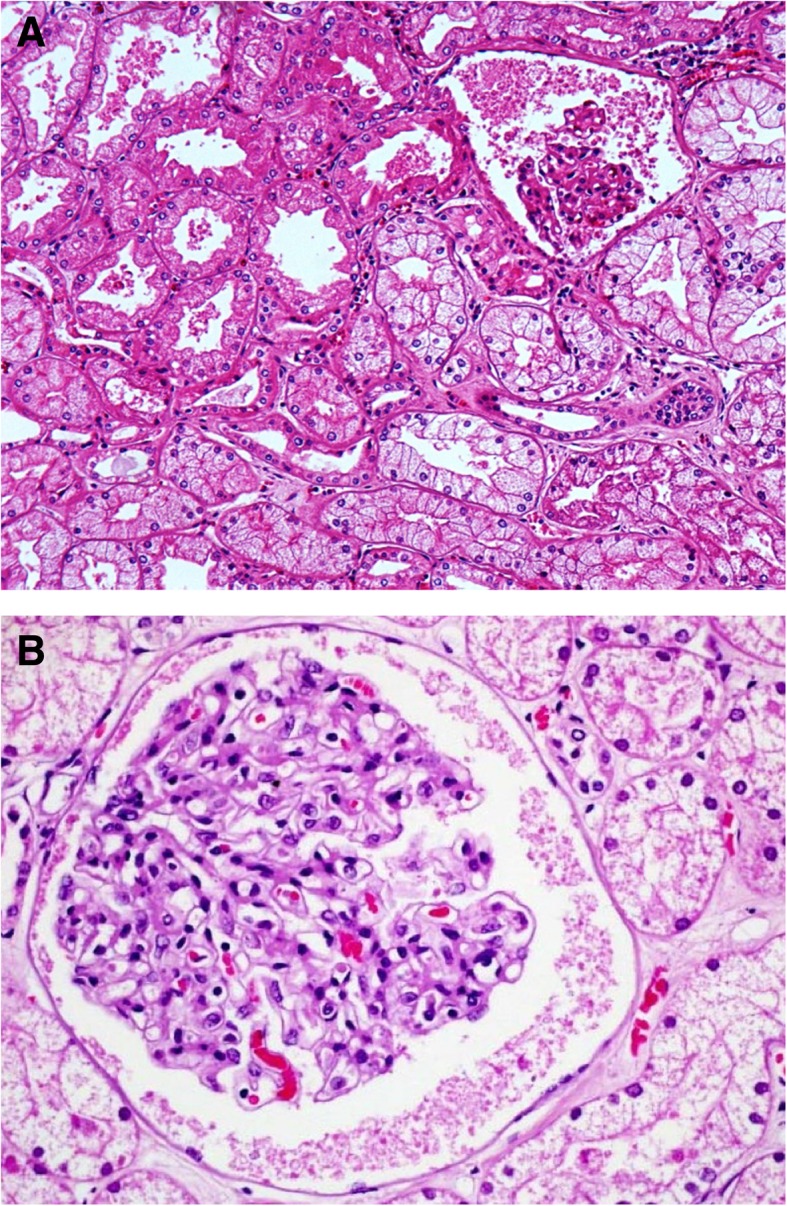
Kidney tissue sample from a patient with sepsis (light microscopy, HE, × 20). **a** Glomerular collapse is observed and signs of acute tubular necrosis. **b** Edema and glomerular and tubular congestion is seen

In summary, results from experimental studies support the interpretation of findings in human studies, indicating apoptosis in combination with nonspecific changes (tubular cell vacuolization, swelling, and brush border injury), and a low prevalence of ATN as the characteristic features of kidney pathology in sepsis (Table 1). Thus structural injury is mild in septic AKI and only partly related to hemodynamic factors (hypoperfusion). The proposed interpretation of these variable findings is that septic kidney pathology results from a complex combination of hemodynamic and inflammatory factors [32, 33]. Typical histopathological changes of sepsis-induced AKI are shown in Fig. 1.

**Table 1:** Summary of findings from kidney tissue samples

	*N*	Acute tubular necrosis	Glomerulonephritis	Tubulointersticial nephropahty	Pyelonephritis	Vascular injury	Apoptosis
Mustonen et al. [17]	57	4 (7.0%)	4 (7.0%)	47 (82.0%)	2 (3.5%)		
Hotchkiss et al. [18]	12	1 (0.83%)					
Diaz de leon et al. [19]	40	20 (50.0%)	11 (27.5%)*			9 (22.5%)	
Sato et al. [20]	6	1 (17.0%)					
Langerberg et al. [23]	117	26 (22.0%)					
Lerolle et al. [24]	19		19 (100.0%)	19 (100.0%)		4 (21.0%)	19 (100.0%)
Takasu et al. [25]	20			15.6 (78.0%)			
Kosaka et al. [26]	1059	184 (17.4%)		915 (86.4%)			953 (90.0%)

Single-case studies are not included in this table. Only studies involving human subjects are considered.^*^Reported in the original study as a combined finding of glomerulonephritis or tubulointersticial nephropathy

### Central nervous system histopathology in sepsis

Sepsis-induced encephalopathy is prevalent in critically ill patients and is associated with increased mortality, and mechanisms involved in its pathogenesis are unknown [34–38]. The pathophysiology of sepsis-induced encephalopathy is incompletely understood [38]. Proposed mechanisms include microorganisms directly invading the central nervous system (CNS), microorganism-derived products entering the CNS [39, 40], metabolic abnormalities affecting CNS function [41, 42], disruption of blood-brain barrier [43–45], changes in neurotransmitter function synthesis and receptorial distribution [46], and impairment of brain circulation and auto-regulation [47, 48]. Thus, knowledge of histopathological changes in the CNS in the context of sepsis is of paramount importance to better understand the pathogenesis of sepsis-induced encephalopathy, determine its reversibility, and define potential preventive or therapeutic interventions. In addition, key manifestations of sepsis, such as cardiovascular dysfunction, could be due to CNS dysfunction [49–52].

The few studies that have analyzed CNS histological changes in sepsis have reported, as the most predominant findings, white matter hemorrhages (case reports of brain purpura [53–55], as well as in 2 of 12 (17%) of patients who died with septic encephalopathy [56]); hemorrhages in 6 cases (26%), hypercoagulability syndrome in 2 cases (9%), micro-abscesses in 2 cases (9%), multifocal necrotizing leukoencephalopathy in 2 cases (9%), and ischemia (23 cases, 100%) in series of 23 cases dying from septic shock, as well as apoptosis and increased vascular iNOS expression [49]; central pontine myelinolysis in 2 of 12 patients with sepsis-induced encephalopathy [56]; metabolic changes in 2 of 12 (17%) of patient with sepsis-induced encephalopathy [56] and in 2 of 23 cases (9%) patients dying with septic shock [49]; ischemic changes in all 23 patients dying with septic shock [49]; and cerebral infarcts in 2 of 12 (17%) cases of sepsis-induced encephalopathy [56] and in three (13%) patients dying with septic shock group [49].

#### White matter hemorrhages and hypercoagulability

Masland and Barrows [53] and Graham et al. [54] presented 1 and 6 cases, respectively, of brain purpura. They were caused by urinary tract infection by *E. coli* in one report [53] and by *Pseudomonas pyocyanea* and *S. aureus* (plus 4 cases with negative cultures) in the other report [54]. They attributed their findings to a generalized Schwartzmann phenomenon (endotoxin-induced vascular thrombosis). Schwenk [55] reported two more cases of purpura cerebri in patients with gram-negative septicemia and proposed that, similarly to acute disseminated encephalomyelitis and acute hemorrhagic leukoencephalitis, purpura cerebri could represent a complication of a generalized Arthus-like reaction (immunocomplex-mediated vasculitis and tissue necrosis) taking place in the wall of the venules of the white matter. One case died of septic arthritis and multiple organ failure, developing coma, generalized seizures and extensive parenchymal lesions, and disseminated focal hemorrhages in the head CT, dying of severe hemorrhagic cerebral lesions and brain edema. CNS macroscopic findings included subarachnoid hemorrhages, bilateral tentorial and slight tonsillar herniations, hemorrhages in the mid-brain and in the upper part of the pons, and numerous petechial hemorrhages in the white matter of both hemispheres. The second case presented pneumonia and peritonitis, developing multiorgan failure, coma, and generalized muscle fasciculations, although the cause of death (e.g., shock, brain death) is not clear form the report. CNS macroscopic findings included swelling, uncal and tonsillar herniations, numerous scattered petechial hemorrhages, restricted exclusively to the white matter in the cerebral hemispheres, and strictly localized to the subcortical zones of the centrum semiovale. The reported microscopic findings of both cases were ball and ring hemorrhages, in whose center a capillary or venule could be found, with vessel wall necrosis and impregnated with a fibrinous periodic acid Schiff (PAS)- and phosphotungstic acid hematoxylin (PTAH)-positive exudate extending into the perivascular area (PAS staining is mainly used for staining structures containing a high amount of carbohydrate macromolecules, such as connective tissues, mucus, the glycocalyx, and basal laminae. PTAH is used to show gliosis in the central nervous system, tumors of skeletal muscles, and fibrin deposits in lesions). Immunohistochemically, scanty deposits of IgG, IgA, and IgM mainly in the macrophages were found.

Jackson et al. [56] examined 12 fatal cases of sepsis-induced encephalopathy. Six of the patients had focal neurologic signs; five had seizures. The level of consciousness varied from drowsiness to deep coma, and electroencephalograms revealed diffuse or multifocal abnormalities. Computed tomographic head scans and cerebrospinal fluid examinations were usually unremarkable. Brain purpura and small white matter hemorrhages were reported in 2 of 12 of patients (17%).

In a more recent study, cerebral hemorrhages were found in 6 patients of 23 patients dying from septic shock (26%), and in none of patients with shock of non-septic origin [49]. One had a large leptomeningeal hemorrhage adjacent to the Sylvian fissure, and the other 5 had disseminated petechial hemorrhages. All 6 patients had clotting disturbances, 5 had thrombocytopenia, and 3 had disseminated intravascular coagulation (DIC). However, coagulation was not significantly different between septic shock patients with and without hemorrhages. Thus, the incidence of cerebral hemorrhage was not related to clotting disturbances.

Hypercoagulability was found in 2 of 23 (9%) patients dying from septic shock [49]. This finding was presented in one case as diffuse intravascular coagulation with multiple fibrinous microthrombi, diffuse small microinfarcts, and hemorrhages; and in another case, as non-bacterial thrombotic endocarditis with multiple distal small embolic infarcts. Clinical DIC and thrombocytopenia was present in both cases. Hypercoagulability syndrome was only found in 1 of the 8 control patients with non-septic shock.

#### Microabscesses

Eight of 12 (67%) patients dying with septic encephalopathy had disseminated microabscesses in the brain at autopsy [56], and blood cultures were positive in 7 of them (88%). Bleck et al. [57] reported postmortem findings in 4 patients that died with septic encephalopathy. None had microabscesses.

In the 23 postmortem studies of cases with septic encephalopathy reported by Sharshar et al. [49], 2 of 23 cases (9%) had microabscesses, and none had positive blood cultures. Septic emboli producing multiple microabscesses were found in 2 cases in the septic shock group but in none of the cases in the non-septic shock group. Gram-negative bacilli were found in the microabscesses, with negative blood cultures [49].

#### Central pontine myelinolysis

Central pontine myelinolysis (CPM) is another finding in septic encephalopathy, as reported by Jackson et al. [56] in 2 of 12 patients, attributed to changes in serum sodium concentration. CPM was not reported in a larger series of patients dying of septic shock [49].

#### Multifocal necrotizing leukoencephalopathy

Multifocal necrotizing leukoencephalopathy (MNL) (whose differential diagnosis is CPM) previously reported in patients with HIV infection or immune suppression, or treated with chemotherapy or radiotherapy for a brain cancer [58–60] was found in 2 of 23 cases dying of septic shock, but in none of the 8 cases dying of shock of other origin [49]. This [49] is the first report of MNL in patients with septic shock. MNL is characterized by multiple small foci of well-defined necrosis disseminated in the white matter of the basis pontis. Other findings in these lesions were loss of myelin, proliferation of lipid-laden macrophages, and swollen and fragmented axons.

The finding that MNL has been associated with a local expression of TNF-α and IL-1β and with high circulating levels of TNF-α, IL-1β, IL-6, IL-8, IL-10, soluble TNF receptor II, and for Il-1 receptor antagonist [60] supports a pathogenic role of circulating cytokines in the development of MNL.

#### Metabolic changes

Metabolic changes were reported in 2 of 12 (17%) of septic patients dying with sepsis-induced encephalopathy [56], characterized by proliferation of astrocytes and microglia. Also, metabolic changes were observed in 2 of 23 cases dying with septic shock [49], characterized by Alzheimer’s type II glia. These changes are nonspecific and may appear in ischemia and many other metabolic abnormalities.

#### Ischemic changes

Ischemic changes were noted in 2 of 12 postmortem studies of patients with sepsis-induced encephalopathy [56]. In the study of 23 patients who had died from septic shock [49], ischemic changes were found in all cases in the five nuclei susceptible to ischemia studied (Ammon’s horn, lenticular nuclei, frontal cortex in the watershed territory between that supplied by the anterior cerebral and middle cerebral arteries, dentate nucleus, and medullary olives). Similar changes were also observed in all 8 patients dying of non-septic shock and (although of less intensity) in 3 of 5 (60%) of control (non-shock) subjects [49]. Cerebral infarcts were found in three (13%) patients in the septic shock group [49]. In Jackson’s study [56], cerebral infarcts were reported in 2 of 12 (17%) cases of sepsis-induced encephalopathy.

All patients in the septic shock group showed ischemic changes in the central autonomic nuclei (CA, SO, PV, LC, V4). Lesions were also found in the non-septic shock group and in the control (non-shock) group, but of much less intensity. Affected neurons showed changes that could be interpreted as indicative of apoptosis, such as shrunken cytoplasm and pyknotic nuclei.

#### Apoptosis

Apoptosis was identified by in situ end labeling (ISEL) and caspase 3 immunostaining in the septic shock but also in the non-septic shock and the non-shock groups, but with a much greater intensity in the septic shock group [49]. Neuronal apoptosis in septic shock did not correlate with neuronal ischemia. There was also no correlation between neuronal apoptosis in the autonomic centers score and duration of septic shock or duration of hypotension, but the intensity of autonomic centers damage (neuronal ischemia score plus and apoptosis score) did correlate with the duration of hypotension.

TNFα and IL-1β and iNOS expression by glial cells was not marked, but intravascular expression of iNOS was significantly higher in the septic shock group than in the non-septic shock group and the non-shock group. Intravascular iNOS expression in the septic shock group was identified almost exclusively in the autonomic centers. Intravascular iNOS expression did not correlate with the duration of septic shock or of hypotension, but did correlate with neuronal apoptosis in the autonomic nuclei [49].

Experimental studies indicate a prominent role of apoptosis in sepsis-induced encephalopathy. In a rat model of CLP-induced sepsis, increased apoptosis (identified by the presence of TUNEL positive cells, caspase-3 immunohistochemistry, and transmission electron microscopy [TEM]) was identified in the median preoptic nucleus, subventricular zone, dentate gyrus, and CA1 and CA3 regions of the hippocampal formation [38].

In summary, the study of brain pathology in sepsis suggests a prominent role of hemorrhage and thrombosis (induced by endotoxin or other bacterial products, by immune phenomena, or by coagulation abnormalities related to sepsis), ischemia, direct bacterial CNS invasion and subsequent microabscess formation, and apoptosis in the pathogenesis sepsis-induced encephalopathy.

We speculate that these findings (hypercoagulability, hypoperfusion, and apoptosis-mediated cell death) could help define therapeutic targets for further clinical testing. In addition, the evidence supporting a role of the CNS in the sepsis-induced cardiovascular dysfunction may also have immediate clinical implications.

### Liver histopathology in sepsis

Liver dysfunction is common in patients with sepsis. Clinical manifestations range from mild elevations of liver enzymes to severe liver failure. Mechanisms involved are not clearly determined and may involve infection, inflammatory mediators, drug toxicity, and metabolic disturbances [61, 62].

Despite the prognostic relevance of liver dysfunction in sepsis, and the lack of knowledge on the precise pathogenesis, there is little information as to the histopathology of liver dysfunction [63–66]. In addition, most studies in sepsis-induced liver dysfunction have been conducted in animal models and in patients after a significant period of time has elapsed from the moment of death to the moment of tissue sampling.

The liver function profile was reported in a series of 57 patients admitted to the ICU with the diagnosis of septic shock [62]. Jaundice was present in 36 (63%) of patients. In 85%, at least one of the liver function tests was abnormal. Post-mortem liver histology was reported in 22 cases. It showed in 16 patients varying degrees of non-specific reactive changes with focal liver cell necrosis, Kupffer cell hyperplasia, and portal tract inflammation; venous congestion; ischemic necrosis; fatty changes; and intrahepatic cholestasis. In the remaining six cases, other characteristic changes were reported: moderately severe cholestasis predominantly perivenular; intracellular bile retention and canalicular concretions; a related inflammatory process with Kupffer cell hyperplasia and aggregates of ceroid-laden macrophages; and dilated cholangioles at the portal parenchymal interface, with swollen epithelium, inspissated strongly PAS-positive bile concretions in many of them, and polymorphonuclear cells in their lumen. Portal/lobular inflammation and/or centrilobular necrosis along with steatosis were the main findings in septic patients. Steatosis, a common finding in the post-mortem liver of septic patients, was moderate to severe comprising 40–80% of the liver parenchyma.

Thus, histological findings in this study [62] were mostly nonspecific (intrahepatic cholestasis, focal liver cell necrosis, Kupffer cell hyperplasia, mild fatty changes, portal tract inflammation) and had been previously reported [63, 67, 68]. Original findings, however, were those in 6 patients in whom in addition to hepatocyte and canalicular cholestasis, bile retention was also evident at the cholangiolar level with abnormalities of the cholangiolar epithelium, conspicuous inspissated concretions within the dilated cholangioles, and a related acute cholangiolitis. These findings, referred to at that time as “inspissated bile syndrome” had been previously described in neonates and children with infection and jaundice and feature cholangiolitis without cholangitis. It was suggested that these morphological changes were responsible for the jaundice observed in patients with infection and sepsis. The proposed mechanisms for these lesions were either a direct effect of endotoxin or hypoperfusion affecting the cholangioles.

Based on this [62] and other studies [63, 64], three histological patterns could be described in patients with sepsis and hyperbilirubinemia: (1) canalicular cholestasis, usually most severe in zone 3; (2) ductular cholestasis with inflammation; and (3) non-bacterial cholangitis associated with the toxic shock syndrome [65].

More recently Koskinas et al. [69] conducted a post-mortem study organ dysfunction sepsis-induced liver histological abnormalities in patients dying after severe sepsis. Needle liver biopsies were obtained within 5 min of death from 15 consecutive patients. The observed histological findings were (1) portal inflammation in 11/15 patients (73.3%), mixed in 8 patients and lymphohistiocytic in 3; (2) centrilobular necrosis in 12/15 patients (80%); lobular inflammation in 10/15 patients (66.7%); hepatocellular apoptosis in 10/15 patients (66.6%); cholangitis in 1/15 patients (6.6%); cholangiolitis in 2/15 patients (13.3%); canalicular cholestasis in 1/15 patients (6.6%); and ductular cholestasis in 2/15 patients (13.3%). Damage to bile duct epithelium was not observed.

Two types of histological injury were defined according to the predominant findings [69]: the “hepatitic” type in 9/15 (60%) patients, characterized by only portal/ lobular inflammation and/or centrilobular, frequently hemorrhagic, necrosis and the “mixed” type (cholestatic and hepatitic), in 6/15 (40%) patients, characterized by different combinations of biliary lesions (bile duct and/or ductular hyperplasia, cholangitis, cholangiolitis), cholestasis (canalicular and/or ductular), portal/lobular inflammation and centrilobular necrosis. Steatosis was observed in 11/15 (73.3%) affecting 5–80% of liver parenchyma. Cholangitis and/or cholangiolitis was observed in a few patients. Additionally, ductular cholestasis, a sepsis-specific hepatic lesion [64, 65], associated with a poor prognosis [70] was identified only in two patients.

The results from Koskinas et al. [69] contrast with results from other studies in which canalicular or ductular cholestasis were predominant findings in septic patients with hyperbilirubinemia.

Another liver complication of septic shock is progressive sclerosing cholangitis [71]. In an evaluation of 29 patients with cholestatic liver disease after septic shock, without evidence of pre-existing hepatobiliary disease, the endoscopic retrograde cholangiopancreatography showed multiple stenoses, pre-stenotic dilatations, and bile ducts partially filled by black-pigmented or necrotic material. Liver biopsies performed in 18 of 29 patients showed fibrosing cholangitis. The endoscopic therapy comprised removal of occluding material, dilation of stenoses, and intermittent stenting if necessary. During follow-up, 19 of the 29 patients died. The median survival was 1.1 years.

**Fig. 2:**
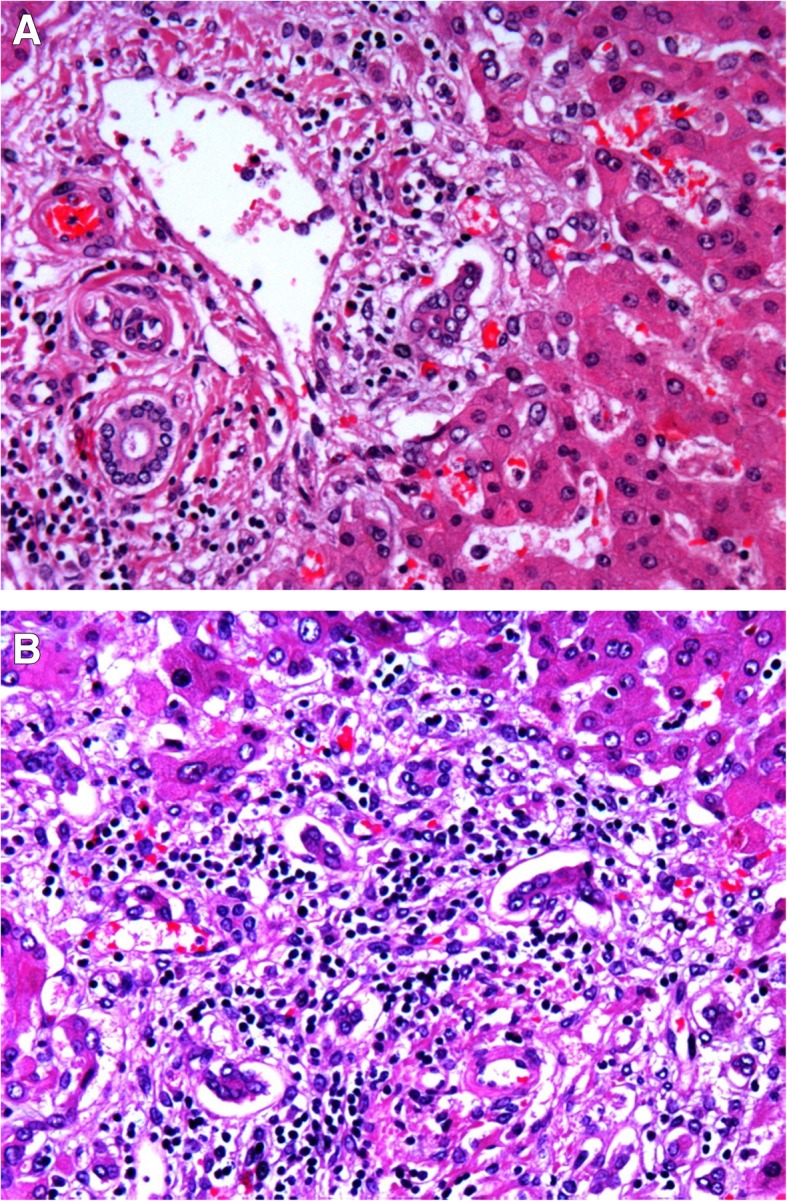
Liver tissue sample from a patient with sepsis. **a** Signs of periportal inflammation are observed. **b** Signs of collangitis

The reported histological findings in patients dying from sepsis [62–65, 69] could be explained by different pathophysiological mechanisms. Mixed (cholestatic and hepatitic) changes could be explained by the effect of endotoxin or a drug-toxicity. Centrilobular hemorrhagic necrosis, another common finding, is a frequent finding in peripheral circulatory failure. Portal inflammation is common in chronic hepatitis. Steatosis has been reported in patients with sepsis [62–64, 69] and can be due to the effect of bacterial toxins that may cause macrovesicular [72] or microvesicular steatosis [73], hypoxia, drugs, and total parenteral nutrition. Liver apoptosis is more common in patients with more severe liver histology [69] and can be explained by the effect of numerous factors, including hypoxia, inflammation, oxygen free radicals, bacterial toxins, and drug toxicity [61, 72, 74, 75]. Intrahepatic cholestasis in septicemia [62] could be attributed to many factors such as circulating inflammatory mediators that alter bile secretion, abnormalities in bile canalicular contraction and ischemia [70, 76, 77]. Typical histopathological changes of sepsis-induced liver dysfunction are shown in Fig. 2.

## Conclusions

In conclusion, the study of histopathological changes in sepsis is of utmost importance to better understand the pathogenesis of organ dysfunction. Although the few identified studies on this area provide a thorough histological description, they fail to relate specific histological findings to the various clinical manifestations of sepsis-induced organ dysfunction. Further studies are needed to establish this relationship. However, the procurement of tissue samples, necessarily from postmortem studies, imposes an unsurmountable difficulty for the design of these studies.

## Abbreviations

AKI: Acute kidney injury
ATN: Acute tubular necrosis
CNS: Central nervous system
CPM: Central pontine myelinolysis
CT: Computed tomography
DIC: Disseminated intravascular coagulation
ISEL: In situ end labeling
MNL: Multifocal necrotizing leukoencephalopathy
PAS: Periodic acid Schiff
PTAH: phosphotungstic acid hematoxylin
TUNEL: Terminal deoxynucleotidyl transferase dUTP nick end labeling
